# Contribution of non-disaster risk reduction non-governmental organisations’ social network building programmes towards community-based disaster risk reduction: Bangladesh context

**DOI:** 10.4102/jamba.v18i1.1952

**Published:** 2026-05-18

**Authors:** Md. Assraf Seddiky, Helen Giggins, Thayaparan Gajendran

**Affiliations:** 1 Department of Public Administration, Shahjalal University of Science and Technology, Sylhet, Bangladesh; 2 School of Architecture and the Built Environment, University of Newcastle, Newcastle, Australia

**Keywords:** community-based disaster risk reduction, non-DRR NGOs, social networks, social capital, Bangladesh

## Abstract

This study aims to explore the contribution of social network-building programmes of non-disaster risk reduction (non-DRR) non-governmental organisations (NGOs) to community-based disaster risk reduction (CBDRR) within the context of the Satkhira district of Bangladesh using social network-building theory. Purposive sampling was employed and semi-structured interviews were conducted with 45 respondents, including beneficiaries of NGOs, NGO officials and local government representatives. Data were analysed using the thematic analysis technique with the assistance of NVivo12 software. The data reveal that non-DRR NGOs contribute to CBDRR by creating bonding, bridging and linking networks that enhance coordination and collaboration at various levels. Bonding networks enhance coordination and collaboration within the community, bridging networks enhance collaboration between different communities, and linking networks enhance collaboration between local and external communities, thereby increasing the availability of resources, technical support and assistance. Such networks enable timely disaster preparedness, response and recovery. Nevertheless, there are several challenges to these functions, such as the diversity of stakeholder interests and an overshadowing of local needs by donor-driven priorities.

**Contribution:**

This study highlights the social network building activities of non-DRR NGOs, their contribution to disaster resilience, and the need to integrate disaster risk reduction (DRR) education into the programmes of non-DRR NGOs. These findings are useful for policymakers, practitioners and other NGOs that intend to use social networks to enhance community resilience. This study also points to the need for further investigation to determine whether these findings are applicable to other settings.

## Introduction

Over the past two decades, the approach to risk assessment and resilience has undergone a dramatic shift, largely because of the increasing frequency and intensity of climate-related disasters (Seidler et al. 2018). While these disasters present challenges globally, developing countries are particularly susceptible to them because their institutions, infrastructure and overall response mechanisms are often inadequate (Hallegatte & Rentschler 2015). In this context, resilience involves more than just technical adjustments or infrastructure enhancement. It is profoundly affected by social and organisational elements, including how communities work together, the robustness of their networks, and the systems they rely on for support. Community-based disaster risk reduction (CBDRR) stands out as a particularly relevant approach, given its focus on grassroots initiatives and the creation of local frameworks that enable collective action before, during and after disaster events (Shaw 2012). Bangladesh in particular provides a compelling example of how these complex factors intersect in the real world. Reflecting on floods in Bangladesh, the country consistently ranks among the most disaster-prone nations globally, experiencing various types of calamities (Mallick, Ahmed & Vogt 2017). Since gaining independence, its economy has been repeatedly affected by floods, cyclones and tidal surges nearly every decade, which have disrupted its economic structure and hindered progress in economic development (Sai et al. 2018).

Bangladesh is the most densely populated country in the world and has prolonged economic dependency on agriculture and fishing (Chowdhury & Jenkins 2018). Since the major disasters of the 1960s, Bangladesh has witnessed the loss of countless lives; however, development progress has also been lost, which in turn has highlighted the need for effective disaster governance (Islam et al. 2021). While the hazard profile highlights the potential dangers, the underlying resilience in the case of Bangladesh is attributed, more than disaster-specific interventions, to the social relations which bind the communities, institutions and resources together (Aldrich 2012). In this widening social context, the significance of activities carried out by non-governmental organisations (NGOs) is paramount. The history of NGOs in Bangladesh is unique. After the birth of Bangladesh in 1971, NGOs began to provide relief and rehabilitation services. However, over time, the range of services has diversified, including grassroots development in the areas of education, health, sanitation, poverty alleviation and women’s empowerment. Currently, there are over 2500 registered NGOs, many of which are well-integrated into the rural population (Seddiky, Giggins & Gajendran 2022b).

Nevertheless, only a small proportion of these organisations focus on disaster risk reduction (DRR). Most of these NGOs are identified in this study as ‘non-DRR NGOs’ entities whose primary objectives are centred on social development rather than disaster management (Seddiky, Giggins & Gajendran 2020). Their contribution to enhancing social resilience, and consequently disaster resilience, is indirect and frequently overlooked in current DRR frameworks. Even when NGOs (non-DRR) do not voluntarily focus on risk management, they significantly strengthen their social networks within a defined community. As a result of carrying out development activities, such as education, health services, savings and credit groups, women’s empowerment, and other social and cultural activities, an NGO enables a framework of social networks characterised by connections on the bonding, bridging and linking social capital levels (Aldrich & Meyer 2015). These networks foster social trust and collaboration at the grassroots level, interlink them across different societal strata, and integrate them with wider social organisations, including governmental agencies, advocacy groups and funding bodies (Lim 2023). In Bangladesh, organisations such as Bangladesh Rural Advancement Committee (BRAC) and Grameen Bank illustrate how sustained community engagement over time builds dense local networks, inter-community collaborative relations, and critical underlying institutional relations that open opportunities for households and communities (Ahmed 2016). These interconnections, reinforced by social networks, are critical for improving community resilience and reducing CBDRR. They enhance the ability to act collectively, mobilise resources and exchange knowledge, which helps communities incrementally, adaptively and transformationally manage social and environmental changes (Aldrich 2019). This situation is still framed by Wolff’s (2021) perception of the Sendai Framework’s focus on community-based efforts. There is still the perception that governments are the only players in disaster management. Disaster risk reduction agencies are the only actors directly responsible for managing DRR, whereas the activities of non-DRR NGOs remain unacknowledged. This is a major blind spot in both scholarly and practical aspects. It is also ethnocentric to consider that the only preparatory work required for developing resilience at the grassroots level is technical in nature.

Bangladesh is an example of such a disconnection. Disaster risk reduction actors still dominate national strategies, yet the absence of these acts and the DRR NGOs’ vertical confinement structures in which the acts operate undermines the desired effectiveness (Jones et al. 2014). However, non-DRR NGOs have the ability to engage with communities for extensive periods, and as a result of their development, they are able to forge long-lasting collaborative partnerships. The social capital that non-DRR NGOs can construct, particularly bonding, bridging and linking, is fundamentally overlooked in the disaster governance framework. While these non-DRR NGOs are particularly important for CBDRR efforts, there is an absence of systematic work that delineates their functions. The limited research on non-DRR NGOs can be attributed to the prevailing tendency in the existing literature to categorise these organisations primarily within the realms of poverty alleviation or development activities (Manandhar & McEntire 2014). There is a discernible bias in disaster research that assumes disaster response to be the sole responsibility of DRR-specialised institutions. This study sought to address this gap by broadening the analytical framework to include the role of developmental work in enhancing resilience and influencing disaster outcomes. This study aimed to assess the role of non-DRR NGOs in Bangladesh in supporting CBDRR by enhancing social capital through bonding, bridging and linking. Instead of viewing them as sidelined, these NGOs are pivotal in community development programmes that nurture the resilience of relational infrastructure. While there is some truth in the assertion that building resilience comes predominantly from formal engagements made to address disasters, it can also be paradoxically constructed from the day-to-day activities of organisations that work to build trust, participation and connectivity in vulnerable settings.

### Social network building towards community-based disaster risk reduction: Theoretical overview

**FIGURE 1:**
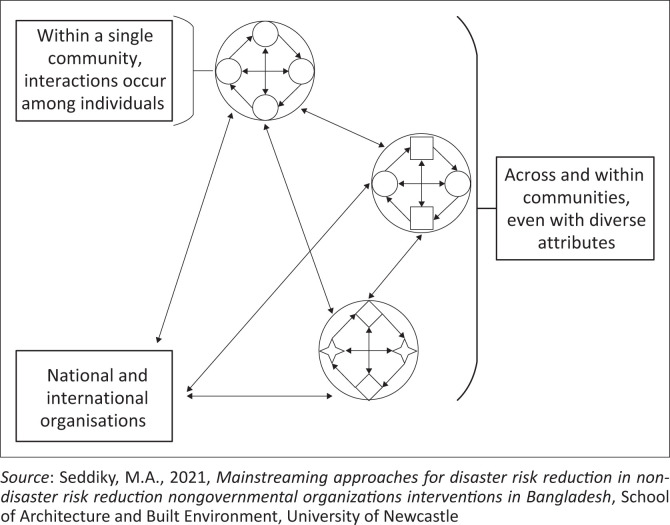
Coordination process of non-governmental organisation.

The increasing frequency and intensity of natural threats have highlighted the significance of community action and individual resilience in reducing the potential impact of any given hazard. The social networks theory of building bonding, bridging and linking networks (Figure 1) offers an interesting perspective for examining community self-organisation and the role of non-disaster risk reduction (non-DRR) organisations in CBDRR indirectly (Pooyan & Hokugo 2025). Instead of viewing social capital as an ambiguous resource, social network theory details the social-structural processes through which people and organisations work together to pool and cultivate trust to enhance preparedness, response and recovery capacity through resource collaboration (Chu & Yang 2020). Each type of network untangled a unique, albeit dependent, aspect of resilience building. Together, these findings illuminate the complex nature of non-DRR organisations’ contributions to community disaster governance. Bonding networks are based on strong, intimate connections that exist in a social group of a given commonality, such as a family, relatives or neighbourhood (Aldrich et al. 2021). These bonds are formed through assistance and trust, especially in crisis contexts. Non-DRR organisations, such as microfinance institutions, women’s saving groups, and cultural and religious organisations, often help strengthen these bonds through participation and joint activities (Rivera 2018). While focusing on employment, schooling and welfare, organisations foster a social context in which trust and collaborative norms emerge among peers. Such frameworks in catastrophic circumstances enable communities to organise and combine resources for timely evacuation, shelter and food provision, or to aid the vulnerable long before formal assistance arrives (Ashida et al. 2017). The effects of strong bonding networks manifest as heightened preparedness and self-help practices that underpin primary disaster responses. However, the exclusive use of bonding networks may foster isolation, inhibiting the acceptance of ideas and interactions with potential collaborators, which is vital for enduring resilience. Conversely, bridging and linking social capital provide avenues for long-term survival and broader community rejuvenation by connecting diverse groups and institutions. Bridging networks cross the outer limits of people’s immediate social circles in every conceivable social, cultural or organisational direction (Liew, Yeates & Lilley 2021).

Although the current literature suggests that they are less dense than bonding ties, bridging networks are important access points for a much wider reach of knowledge, innovation and opportunities. Many non-DRR NGOs assume bridging ties by connecting communities to peer networks, civil societies and professional organisations (Jones et al. 2014). For example, agricultural cooperatives that bring together farmers from villages go beyond economic ends to promote the sharing of climate change adaptation and disaster risk-preparedness strategies. Health and social youth associations, within their primary spheres of interest, often link with local disaster management committees to raise cross-social group disaster awareness and organisational skills. With respect to CBDRR, bridging networks imply a wider resource availability. Communities are exposed to various coping strategies and collaborations (Shaw 2012) that strengthen bridging ties and resource integration; such efforts represent an intentional move toward in situ culturally relevant strategies and the endorsement of regional collaboration (Sohn 2014).

Linking networks finishes the illustration by bridging communities to institutions, authorities and individuals with formal power and resources. Compared with bonding and bridging ties, which are horizontal, linking networks are vertical and include connections with governmental arms, patrons and global bodies (Page-Tan 2021). These ties are largely facilitated by non-DRR organisations, which, because of their focus on other sectors, such as health, education or advocacy, end up interacting with formal structures (Karso, Hardi & Baryalai 2025). For example, when a health NGO partners with a public authority to carry out a vaccination campaign and, in the process, helps position communities access healthcare support in the aftermath of a disaster (Aldrich & Meyer 2015). Linking networks incorporate local concerns into wider disaster governance frameworks, thus enabling disaster-affected communities to access vital infrastructure, warning systems and relief funds (Hermansson 2016). These ties are not without danger, which includes fostering dependency and elite capture, in which case the most marginalised are likely to be overlooked. Bonding, bridging and linking networks together create a structural layer of resilience that is key to CBDRR (Pfefferbaum, Van Horn & Pfefferbaum 2017). Bonding ties provide social cohesion and rapid mobilisation, bridging ties widen adaptive capacities through community interchanges, and linking ties integrate community actors into formally recognised disaster governance systems (Partelow 2021). The contributions of organisations not directly involved in DRR are nuanced, yet significant. Embedding resilience into activities such as managing livelihoods, education and healthcare systems reinforces various social networks vital during times of crisis. In the short term, a community that relies on bonding social capital can exhibit unity after a disaster only to face the risk of social isolation later during the recovery process. In contrast, a community that possesses strong bridging and linking social capital but is paradoxically deficient in bonding social capital has easy access to external resources but lacks internal cohesion to mount collective action (Chu & Yang 2020). The interplay of all three dimensions – bonding, bridging and linking – leads to the convergence of resilient and inclusive forms of resilience in a community.

This synthesis is a case in terms of the practical value of social network building theory in relation to CBDRR. By transcending disaster-specific institutional frameworks and inquiring into the social and organisational ties that underpin resilience, the theory makes a strong case for the circumstantial, yet often overlooked, contributions of non-DRR organisations. Their constructive impact on the bonding, bridging and linking network tie framework completes the theoretical perspective of embracing a multidimensional form of resilience that is community-centred and institutional.

**FIGURE 2:**
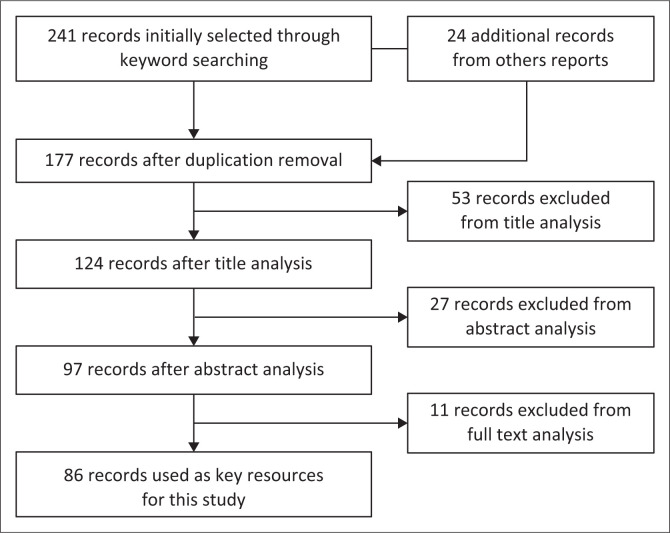
Systematic literature review process.

This research utilised Google Scholar and Scopus as essential tools to meticulously identify and examine articles, books, chapters, reports and conference papers that explored the role of NGOs’ social network-building initiatives in strengthening CBDRR. To prevent reference duplication, we employed an EndNote reference manager. Figure 2 provides a summary of the studies selected for this study. The mesh terms used to select the literature included non-DRR NGO, social networks, CBDRR and social capital.

## Research methods and design

This study followed a qualitative research approach grounded in the social constructivist paradigm to examine the contributions of non-DRR NGOs’ social network building programmes to CBDRR. This approach was employed to capture participants’ perceptions, attitudes, experiences and interactions in real-life situations. To understand the impact of non-DRR NGOs’ social network theory on fostering bonding, bridging and linking for effective CBDRR, a qualitative approach is essential. As noted by Mercer (2006) most research on NGOs in the development sector has employed qualitative methodology.

**FIGURE 3:**
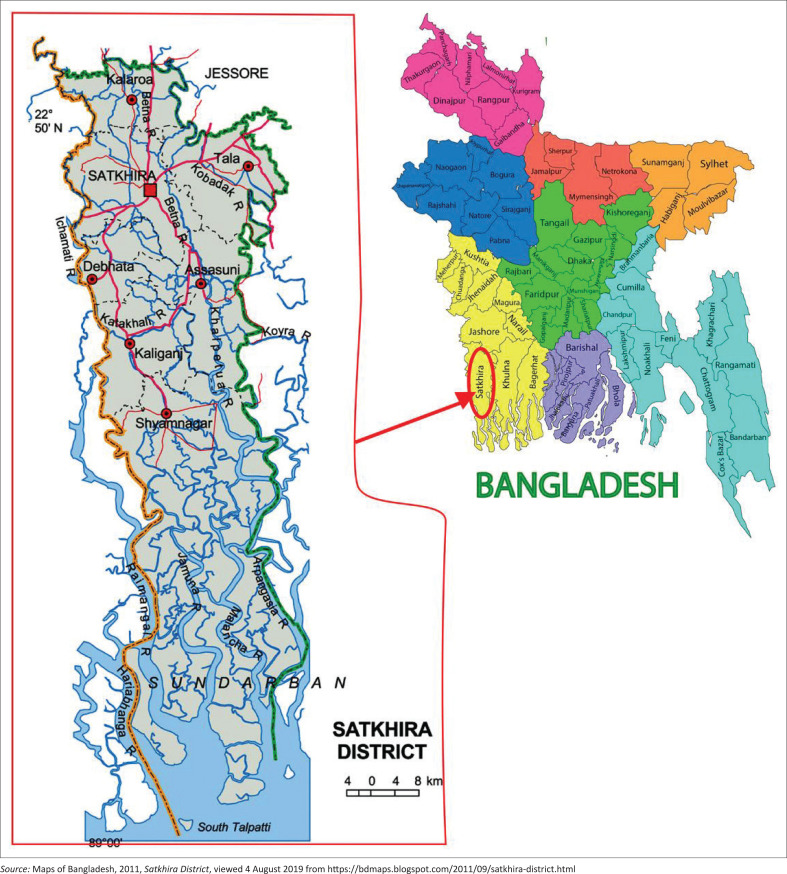
Study area.

This study focused on selected areas within the Satkhira district, located in the south-western coastal region of Bangladesh (Figure 3). The district houses approximately 1.9 million residents, with nearly half of the population involved in fishing-related occupations. Aside from being part of the broader southern coastal region, Satkhira is particularly vulnerable to natural hazards (Pal et al. 2016). Its proximity to the Bay of Bengal increases its exposure to hazards, such as floods, tidal surges and cyclones. Several factors contribute to this vulnerability, notably the district’s low-lying tidal plain geography and the underdeveloped socio-economic conditions.

These recurrent natural events frequently result in increased salinity and an increase in the incidence of waterborne diseases. Sarker et al. (2019) observed that following such calamities, salinity in both fresh and surface water tends to rise, which is accompanied by a higher incidence of waterborne transmissible diseases. Elevated salt levels in water impact well-being and are directly linked to health issues such as high blood pressure, toxaemia, skin disorders, acute respiratory syndrome, and diarrhoeal diseases (Talukder et al. 2016). Most Upazilas, which are second-tier local governments in this district, have endured prolonged hardships because of flooding and waterlogging, resulting in considerable casualties and economic damage (Alam, Chakraborty & Islam 2019). For instance, the recent cyclones Sidr, Aila, Fani, Bulbul, and Amphan caused economic losses of approximately USD 32.75 million, resulting in 82 deaths, injuring 1,541 people and damaging approximately 170 000 houses in the region (Seddiky 2021).

Numerous domestic and local NGOs have been actively engaged in this area, focusing on efforts to improve the community’s well-being. Consequently, the researcher gained first-hand experience with the NGO personnel and local communities affected by these conditions. In qualitative research, determining the sample size lacks strict guidelines, but the process continues until data saturation is achieved (Tutar, Sahin & Sarkhanov 2024). Although an excessively large sample size could lead to unnecessary repetition, the sample size should be sufficient to uncover key insights (Guetterman 2015). Malterud, Siersma and Guassora (2016) correctly observed that specific numbers are not recommended for subjective research, but they suggest looking at sample sizes from previous studies with similar designs where saturation was achieved and using a number within that range. Therefore, the concept of saturation determines the sample size in this study.

Using a purposive sampling approach, the researcher chose 46 participants for interviews from the study region, which encompassed beneficiary communities, NGO officials and local government representatives. This research targeted 10 NGOs that have been engaged in community development efforts in the Satkhira district for a period of no less than 10 years (established before 2003), the individuals benefiting from these community-driven programmes led by the chosen NGOs, and the leaders across different tiers of the local government (Table 2). In (2015), Chanhthamaly conducted a study in Laos by selecting participants from local government, NGOs, and the community to explore the functions of NGOs. This selection was made because participants needed to possess relevant expertise and background, which were essential for providing insightful responses to the subject of the investigation. The reason for using purposive sampling was to find information-rich cases that aligned with the aim of the research, which in turn helped achieve a deeper understanding. Following these principles, purposive sampling was used to select participants who had significant experience with NGO community-based projects and were willing to share their views on the issues being studied. It should be highlighted that every participant in this study was at least 18 years of age. On average, those who were interviewed had been involved in NGO initiatives for approximately 2 years and 6 months (Table 1). Their extensive and functional involvement in non-DRR NGO projects is crucial for enabling researchers to achieve their goals.

**TABLE 1:** Socio-demographic profiles of individuals (interviewed) benefiting from non-governmental organisations in the Satkhira district.

Attributes	Frequency distribution (*n* = 18)	% (Approximate)
**Age categories of participants (years)**		
18–25	01	6
26–35	05	28
36–45	04	22
46–55	03	17
56+	05	28
**Gender breakdown of respondents**		
Male	05	28
Female	13	72
**Educational background of respondents**		
No formal education	02	11
Primary school level	08	44
Middle school level	03	17
High school level	04	22
Other qualifications	01	6
**Employment status of respondents**		
Agriculture	05	28
Entrepreneurship	04	22
Home maker	09	50
Salaried employment	00	0
Other occupations	00	0
**Monthly income range of respondents**		
5000–10 000	04	22
10 000–15 000	08	45
> 15 000	06	33
**Marital background of respondents**		
Wedded	16	88
Single	01	6
Maritally dissolved	00	0
Widowed	01	6
**Years of engagement**		
1–3	07	39
3+	11	61
**Residential patterns of respondents**		
Brick-built house	10	56
Mud and Kaccha house	08	44

**TABLE 2:** Respondents selected for this study.

Category of respondents	Role of affiliation	Male	Female	Number
Recipients of community-based support	Individuals receiving services from NGOs	13	5	18
NGO staff members	NGO leaders and programme personnel	17	6	23
Representatives from local administrative units	UNO, DC, UP Chairman	5	0	5
Excluded prior to data analysis	-	-	-	−1
**Subtotal**	-	-	-	**45**

NGO, non-governmental organisation; DC, district commissioner; UNO, Upazila Nirbahi Officer; UP, Union Parishad Chairman.

The research involved key individuals from various tiers of local government, such as the District Commissioner (DC), Upazila Nirbahi Officer (UNO), and Union Parishad Chairman (UP), as the key respondents. All local office representatives were invited to participate in quarterly meetings led by the DC for NGO coordination. This involvement equipped them with a thorough understanding of the local initiatives undertaken by NGOs.

The researcher decided to use a semi-structured interview line of questioning because of its capacity to delve into the subject, which in turn minimises the chances of any form of bias. Originally, the sample included 46 participants; however, one individual opted to withdraw from the study without showing any reason. The analysis of the interview scripts of 45 participants formed the foundation of this study. The researchers meticulously extracted and organised both textual and visual data from the project documents and reports, focusing on the central questions of the research project through repeated document reviews.

### Data analysis

In this study, the researcher utilised thematic analysis to scrutinise the primary data using NVivo12 software (QSR International Pty Ltd, Doncaster, Victoria, Australia). Thematic analysis is celebrated for its versatility in qualitative research, making it a favoured approach for exploring diverse investigative goals and subject areas, and providing insights that quantitative methods often fail to capture (Castleberry & Nolen 2018). Moreover, non-numerical data analysis is primarily inductive, enabling patterns and themes to arise from the data instead of being guided by a predetermined hypothesis, as outlined by Giesen and Roeser (2020). They describe six steps in the qualitative data analysis process (Table 3). Various de-identification codes were assigned to different participant groups such as NGO officials (NOP), local government bodies (LGP) and community beneficiaries (CBP).

**TABLE 3:** Data analysis process.

Step	Process	Description
1	Collect primary data	Accumulate field notes, documents, images and other pertinent materials.
2	Organise the data	Arrange and classify the gathered data systematically for easy access and review.
3	Familiarisation	Immerse yourself in the data by repeatedly reading notes and listening to recordings.
4	Data coding	Categorise the data using open coding, selective coding and pattern coding techniques.
5	Categorisation and connection	Explain and link coded data based on similarities and relationships.
6	Theme analysis and interpretation	Examine and interpret overarching themes to uncover deeper meanings within the data.

*Source*: Based on Giesen, L. & Roeser, A., 2020, ‘Structuring a team-based approach to coding qualitative data’, *International Journal of Qualitative Methods* 19, 1609406920968700. https://doi.org/10.1177/1609406920968700

The researchers employed a two-cycle coding strategy, which included both structural and provisional coding techniques. Structural coding, often referred to as open coding, is an inductive method originating from the data itself. In contrast, provisional coding is shaped by pre-existing concepts and theories (Hemmler et al. 2022). The researchers engaged in initial coding (open coding) to uncover unique concepts for categorisation, which facilitated the progress of their study. During the subsequent stage, termed selective coding, the process involved refining and merging categorised data, which ultimately led to the formation of theoretical insights that were used to select and integrate organised data categories, ultimately leading to the formulation of theories. The process evolved to a more abstract level to develop a case narrative (Table 4). A document review was carried out to better comprehend the contextual application and the significance of individual participants.

**TABLE 4:** Themes, selective codes and open codes on non-governmental organisations’ network-building programmes.

Emerging themes	Selective coding	Open coding
Programmes developed by NGOs to build bonding social networks play an essential role in disaster relief, as they contribute to reducing the risks, vulnerabilities, and exposure linked to hazards.	Bonding social network	Flow of information Awareness raising Interpersonal relations Collective efforts Fairness and trust
NGO initiatives focused on fostering bridging social capital are essential for providing immediate aid during emergencies and supporting the long-term recovery of impacted individuals.	Bridging social network	Institutional collaboration Local media Communal efforts Public-private partnership Structural loopholes
The diverse aspects of linking social capital allow communities impacted by disasters to obtain emergency assistance, receive ongoing livelihood support, and ultimately enhance their ability to withstand future disaster risks.	Linking social network	Bottom-up relationship Relation with people in power Relation with organisation in position National and international attention

NGO, non-governmental organisation.

### Ethical considerations

Ethical clearance to conduct this study was obtained from the University of Newcastle, Human Research Ethics Committee (No. H-2019-0068).

Prior to the interviews, the researcher prepared and provided informed consent to the participants. These forms included details about the purpose of their involvement, the criteria for participation, the rights of the participants, the method of contact, and the length of each session, which was approximately 1 h. The University of Newcastle mandates that ethics approval be obtained from the Human Research Ethics Committee (HREC). These procedures require that the investigator adhere to certain ethical standards. The ethics application for this project, numbered H-2019-0068, was approved on 12 June 2019. Ethical principles were upheld throughout the data collection and analysis phases to protect the integrity of both the participants and the researcher.

## Findings and discussion

Both non-governmental entities and communities identified them as beneficial and targeted, and fostered their social relations during weekly meetings and training, as well as through participation in discussion panels of community associations. Social capital is used to increase the ability of the community to request assistance, communicate, gather information and resources, facilitate the linkage of the government and other local and national organisations, and collaborate on DRR. Beneficiaries have created and fostered three different types of social capital: bonding social capital among community members, bridging social capital between community-organised groups, and linking social capital with external organisations. All were successful in reducing disaster risk. The next section analyses emerging issues regarding the role of non-DRR NGOs in social networking and in supporting CBDRR:

### Non-governmental organisations’ bonding social network building programmes play an essential role in disaster relief, as they contribute to reducing the risks, vulnerabilities and exposure linked to hazards

Non-governmental organisations play a crucial role in fostering and strengthening connections between community members through local associations. Non-governmental organisations have empowered CBP by offering training in skill development and raising awareness of socio-economic and ecological concerns. The participants then shared the insights and learning they gained with others in their groups, which not only reinforced strong bonds, but also encouraged collective action to address ecological and socio-economic challenges. During disasters, local communities rely primarily on mutual support and collaboration to address the difficulties they face (Partelow 2021). Initially, friends, family and neighbours stepped in to provide rescue, relief and repair of damaged homes. Consequently, social bonds contribute to reducing a community’s vulnerability and risk during disasters through joint effort. Prior to any flood, cyclone or tidal surge, social capital built through these bonds aids in gathering critical information for preparedness and preventive measures, thus motivating the community to engage in safety-focused activities. As expressed by the participants:

‘We are invested in helping beneficiaries share information as quickly as possible with their family members, relatives, and neighbours. Local people received warnings ahead of time, unlike on previous occasions where information was disseminated just before the event. A network of local volunteers quickly spreads disaster signals around the community to enable people to obtain well-prepared.’ (NOP12 A, male, 42)

Another study by Kim and Hastak (2018) produced comparable findings, given the government’s limited ability to manage disaster aftermath; community members, families and neighbours often act as initial responders, playing a vital role in early disaster relief efforts. Historically, communities have received warnings shortly before events because of inadequate communication systems, leading to poor disaster preparedness and significant damage. Non-governmental organisations improve preparedness through group-based strategies within their networks. Beneficiaries of NGOs receive alerts from officials and promptly inform their families, relatives and neighbours, enabling them to prepare and relocate safely. They also assist vulnerable groups such as people with disabilities, the elderly, pregnant women and children in reaching cyclone shelters, thereby enhancing disaster readiness by reducing the community’s exposure and vulnerability in socio-economic, physical and environmental terms (Lee et al. 2022). Non-governmental organisation community associations have gained acceptance owing to their expertise, collaborative efforts and developmental roles, encouraging organised responses to potential threats. A beneficiary participant noted:

‘After announcing disaster signals, we request members of the community to get prepared to move to safety along with their important documents and cattle. Our team members also helped bring pregnant women, children, the elderly, patients, and the disabled to the shelter camps. We also brought oral saline, dry food, and torch lights to shelter camp.’ (CBP4, female, 33)

Moreover, during disasters, when the trafficking of women and children increases, association members collaborate with communities to act as guardians, ensuring the security and welfare of women and children in emergency storm shelters (Seddiky, Giggins & Gajendran 2022a). The community has come to trust and respect the organisation’s members for their expertise and collaborative efforts. Trust plays a crucial role in reinforcing interpersonal connections, which, in turn, enables the swift dissemination of information, cohesive and sensible decision-making and enhanced teamwork. The research by Depping et al. (2016) reported consistent results, showing that trust between individuals is crucial for successful teamwork because it enhances communication and collaboration among team members. This synergy is instrumental in reducing hazard risk, susceptibility and exposure, thereby strengthening the mitigation and preparedness efforts.

One beneficiary remarked:

‘I will say that the NGO’s main achievement is our unity. NGOs have created different groups and similarities (associations) among the different types of beneficiaries. We are now well organised, and we handle the situation collectively. We have developed a relationship among community members, and we use this bonding to help each other in any crisis.’ (CBP9, female, 29)

Sanyal and Routray (2016) reached similar conclusions, emphasising that, within the action priorities of the SFDRR, community-level associations are valuable for their accepted capacity to foster member cooperation, share knowledge and strengthen communities to implement self-driven localised hazard mitigation plans. Similarly, Ahmad and Afzal (2022) in their research found that in disaster-prone areas, communities are much more prepared for potential disasters, primarily as a result of strong community-level associations and enhanced perceptions of equity and trust. Overall, in non-DRR NGOs, bonding network activities within communities help strengthen people’s social ties and build cohesion in times of adversity, which is vital in CBDRR.

Non-DRR NGOs also play an important role in community development. They mobilise and promote self-help, savings, and credit in cooperative societies, cultural groups, and volunteer groups. This builds trust, mutual support and collective actions. Such networks and groups provide coping mechanisms during disaster exhaustion and facilitate the resilience of social capital, which eases the processes of sharing intensive information on social order during disaster preparedness and recovery. In this regard, the form of bond non-DRR NGOs is vital in promoting social communities, responding to disasters, and supporting the need for community resilience:

### Bridging social capital building programmes of non-governmental organisations contributes to emergency relief and long-term recovery of the affected people

In the realm of CBDRR, creating bridging networks plays a vital role in uniting various groups and promoting inter-community cooperation as well as ensuring the flow of information, resources and assistance during critical times. Non-governmental organisations’ training sessions and gatherings help to build amicable ties between people from different areas and communities. Through active collaboration and partnerships with local entities, such as educational institutions, faith-based organisations and individuals from different communities, NGOs have created a wide-ranging and resilient network that can be counted in times of crisis (Pal & Shaw 2018). These local organisations include a diverse range of socio-economic groups that enhance the effectiveness of disaster preparedness and management by improving the quality of responses, resources and local expertise.

By engaging NGO beneficiaries in training sessions and meetings held in government and private offices, the scope of positive interactions and collaboration with people from various communities has broadened. By incorporating grassroots organisations into their regional initiatives, NGOs foster stronger connections with these local entities and the communities they intend to support. The interconnectedness between locally rooted organisations and their counterparts in various regions expands collaborative efforts, aiding immediate assistance and the sustained rebuilding of communities in high-risk areas. This also bolsters their ability to reduce risk and minimise susceptibility to harm (Acosta et al. 2018). In the study region, when severe storms such as Aila, Sidr, Foni and Mahasen occurred, the affected communities obtained substantial support, including financial support, medical supplies, food assistance, materials for rebuilding shelters and access to clean drinking water. A robust network of local groups and organisations significantly contributes to minimising disaster vulnerability and improving preparedness.

As expressed by the participants:

‘NGOs are also creating social capital for strengthening community capacity to reduce disaster risks. They are developing ties with business communities, teachers, farmers, imams, pastors, and other groups from different socioeconomic backgrounds to work together. This helps spread information about the affected community’s actual situation and enables them to get external help.’ (LGP2, male, 55)

Comparable results from Sanyal and Routray (2016) suggest that following the 1985 earthquake in Mexico, networks of individuals and civic-based organisations across different areas came together to assist those affected, demonstrating a strong bridging connection. Kristian and Ikhsan (2024) discovered that when community members were introduced to and engaged with local institutions, they facilitated their positive adaptation and response to crises. The participation of both mainstream and online communication platforms in community forums, on-site demonstrations and public gatherings has significantly expanded the reach of these initiatives and has played a crucial role in enhancing inter-community connections. The local press, trusted for its wide reach, serves as a key source of information, disseminating news, raising awareness and soliciting support from affected communities before and after disasters (Ogie et al. 2022).

In response to early disaster warnings from the media, many individuals evacuated to safer locations, using dried goods, essential records and livestock as part of their emergency readiness efforts. Additionally, numerous individuals and organisations nationwide responded to media calls for assistance, providing resources such as water pumps, communication devices, inflatable rafts, packaged meals, utility carts, and assistance with housing restoration to alleviate both immediate and long-term vulnerabilities, thereby accelerating disaster mitigation and preparedness efforts. Non-governmental organisations’ community-level partnerships helped bridge the gaps between various groups and communities by addressing structural weaknesses, which are vital for reducing disaster risk.

One beneficiary participant said:

‘We have also developed good relations with people from other districts united by the NGO serving in our communities. We were introduced during district- and subdistrict-level meetings and training. Contact information was also available during emergencies. This has proven to be helpful in the past. For instance, during the cyclone in 2007, NGO members from other districts collected a lot of relief supplies and brought them to the affected people of this area.’ (CBP18, Female, 37)

Similarly, Ganoe, Roslida and Sihotang (2023) highlighted in their research that local media plays a crucial role in preparing communities for disaster threats by effectively raising awareness and initiating preparedness and recovery actions. The majority of the services provided by community-level associations supported by NGOs rely on voluntary involvement. Irrespective of cultural or demographic background, participants are committed to fostering societal development and emergency preparedness through charitable activities. They act as liaisons between local communities and main crisis management bodies, customising solutions to meet specific community needs and facilitating communication between at-risk populations and authorities (Shah et al. 2023). Non-governmental organisations empower the communities they serve by forging partnerships and fostering relationships across disparate scales to enhance community disaster resilience (Azad et al. 2019). The resilience witnessed in many communities, where NGOs operate, can be attributed to their ability to construct networks comprising local institutions, voluntary organisations, the press and the populace. These networks aid in integrating the various phases of disaster preparedness, response and recovery, ensuring that the community’s disaster response and recovery mechanisms are optimised.

### The diverse aspects of linking social capital allow communities impacted by disasters to obtain emergency assistance, receive ongoing livelihood support, and ultimately enhance their ability to withstand future disaster risks

In CBDRR, the integration of local social networks with influential figures, government entities and international organisations enables these networks to access essential information, resources, financial disaster management and ongoing post-disaster support. Non-governmental organisations are essential for orchestrating efforts across the local, national and international spheres to support communities during extended recovery periods. Once immediate relief supplies are provided by local entities and communities, they must rely on external organisations for sustained recovery support, which largely hinges on organisational and community linkage capabilities (Abiddin, Ibrahim & Abdul Aziz 2022).

NGOs build strong alliances with government officials and beneficiaries to raise awareness of the potential risks and challenges, thereby enhancing their preparedness. They often engage with global donors to execute local projects because a significant portion of their financial support is derived from these sources (Mikeladze 2021). Non-governmental organisations obtain financial support from global aid agencies to assist individuals in crisis responses and rebuilding efforts. They also receive support from national donors to aid communities by building temporary shelters, repairing homes and providing health services, all of which aim to reduce socio-economic and physical vulnerability, and enhance disaster preparedness. One participant stated:

‘We not only organise the community but also communicate and coordinate with national and international organisations to let them know the actual situation and sufferings… to attract the attention of the donors so that they can provide support regarding relief and rehabilitation.’ (NOP6, male, 59)

Non-governmental organisations offer beneficiaries the opportunity to engage in both national and international communities. Through community-level associations, NGOs facilitate interactions with NGO officials, local government representatives, elected officials, and occasionally donor representatives during training sessions, meetings, or programme orientations, fostering strong relationships. Similarly, Yan, Lin and Clarke (2018) stated that in collaborative efforts across different sectors, NGOs can take on various roles, such as consultants, intermediaries, communicators and advocates, enabling them to utilise their networks to drive significant social transformations. Non-governmental organisation beneficiaries use mobile phones to reach out to relevant individuals and organisations for assistance in disaster-stricken areas. An extensive social capital network empowers communities affected by disasters to receive immediate support, such as emergency aid and crisis intervention, and ongoing assistance, including innovation, agricultural inputs and livelihood support. This finding is consistent with Page-Tan (2021), who argues that technologies enhance coordination, facilitate the spread of information, and boost community involvement, leading to more effective disaster response and recovery operations. This network not only garners increased attention from both domestic and global stakeholders, but also bolsters local resilience, aids in risk mitigation, and enhances readiness for future challenges. Similarly, Quader et al. (2023) provide evidence that NGOs contribute to the development of linking social capital by working alongside influential actors or institutions to execute DRR projects within communities.

One of the participants said:

‘Our NGO facilitated connections with governmental and international bodies, equipping our community with the necessary resources and support to enhance disaster preparedness and recovery efforts.’ (CBP8, female, 45)

In essence, linking networks developed by NGOs strengthens CBDRR by aligning local requirements with national and global resources, promoting collaborations with relevant partners, and providing access to financial, technical and institutional assistance. These networks also address governance and coordination issues to bolster community robustness and risk reduction, support recovery and adaptability, and enhance responses to future disasters. Nevertheless, efforts to collaborate across multiple levels and organisations have introduced complexities in forming an efficient network. Close-knit groups within these systems can leverage their closeness to authorities and policymakers to advance their own agendas (Valdez, Vassallo & Braunstein 2023). Non-governmental organisation coordination initiatives frequently neglect elected officials as they are not directly obliged to them. This oversight is misguided, given that civic-elected leaders are vital in their local areas and can assist NGOs in crafting and executing initiatives that truly benefit the local population.

As for the local government, the issuance of permits and licences to NGOs intending to undertake community projects has always been performed in close collaboration with NGOs. Non-governmental organisation representatives claim that there are several obstacles to gaining permission from civic authorities for community projects, especially the illegal payment of bribes. Therefore, incorporating hazard vulnerability into NGO activities and preserving their standards is very difficult. Local authorities openly solicited bribes, while civic leaders pressured NGOs to prioritise distributing aid to their political patrons. Such cooperation has bred cross-interest among NGOs and local governments.

In summary, NGOs have been able to utilise their social capital to form networks on regional and global scales, which have, in turn, enhanced the strategies of preparedness, mitigation and prevention for the communities they serve. Nonetheless, there are occasions when conflicts of interest among local stakeholders disrupt these cooperative endeavours, ultimately increasing the exposure of communities to disaster risks.

## Conclusion

### Policy implications

Bangladesh has a fascinating development circle. Although a growing population is an asset for economic progress, the country’s development indicators are not encouraging because of pervasive poverty and ongoing natural hazards. Natural calamities, such as tremors, storms, inundations and landslides, have uneven impacts and greatly affect the bulk of the population that does not have proper resources and infrastructure. Grassroot interventions in under-represented populations are a hallmark of the transformational narrative, as NGOs strategically collaborate with government-sponsored programmes. Although DRR is crucial, it is important to recognise that numerous NGOs prioritise actions related to response and recovery following a disaster rather than focusing on preventive strategies. Concerns have been raised regarding the effectiveness of these current approaches, considering the DRR principles that, if adopted, could improve the efficiency and effectiveness of these approaches. At the grassroots level, NGOs foster the formation of networks that generate trust and collaboration among members, thereby facilitating collaboration to achieve shared goals. They help to establish both formal and informal communication systems that operate within and beyond the community.

Their local branches encourage members to actively participate in group discussions, information-sharing sessions and training workshops, which helps strengthen bonding, bridging and linking networks. Such networks are useful for pre-positioning resources to mount a swift response to a disaster and facilitate long-term recovery assistance. In addition, these networks improve communities’ preparedness and capacity to reduce risks by increasing access to technology and funds for risk reduction and community development overseas. By facilitating the sharing of specialised technical insights, training and hands-on experience, such coordination plays a crucial role in fostering local development and mitigating the community’s susceptibility to hazards and risks.

Building relationships with remote communities and organisations enhances the community’s access to knowledge, information and finances, which improves community-enduring relief and reconstruction efforts and thus strengthens the community’s resilience. Non-DRR NGOs have the potential to create robust social networks for disaster risk management by fostering meaningful collaboration among local governments, development NGOs and DRR-focused organisations. It is crucial to ensure that these social network programmes align with the national and local DRR strategies. Incorporating DRR education into community-focused social network programmes can empower communities to identify risks and mobilise effectively during disasters. All stakeholders should prioritise holistic development over personal interests by leveraging social networks initiated by non-DRR NGOs. Special consideration should be given to vulnerable groups within community networks, such as women, children and the elderly with disabilities. This study’s exploration of non-DRR NGOs’ social network-building programmes and their contribution to CBDRR provides valuable insights for policy makers, NGOs, communities and practitioners. These findings can help strengthen social capital development efforts through non-DRR NGO programmes to enhance community resilience. Future research could apply institutional theory, dependency theory and legitimacy theory, involving a broader range of participants, to examine the effectiveness of NGOs’ social network-building programmes in enhancing CBDRR. This approach would contribute to validating or refuting the conclusions drawn from this investigation, while also filling gaps in knowledge within related fields. The research findings are grounded in the context of Satkhira district, which is representative of many rural areas in Bangladesh. However, caution must be exercised when applying these results to economically advanced countries.
